# HIV-1 Infection of Bone Marrow Hematopoietic Progenitor Cells and Their Role in Trafficking and Viral Dissemination

**DOI:** 10.1371/journal.ppat.1000215

**Published:** 2008-12-26

**Authors:** Aikaterini Alexaki, Brian Wigdahl

**Affiliations:** 1 Department of Microbiology and Immunology, Drexel University College of Medicine, Philadelphia, Pennsylvania, United States of America; 2 Center for Molecular Virology and Neuroimmunology and Center for Molecular Therapeutics and Resistance, Institute for Molecular Medicine and Infectious Disease, Drexel University College of Medicine, Philadelphia, Pennsylvania, United States of America

## Abstract

Patients with HIV-1 often present with a wide range of hematopoietic abnormalities, some of which may be due to the presence of opportunistic infections and to therapeutic drug treatments. However, many of these abnormalities are directly related to HIV-1 replication in the bone marrow (BM). Although the most primitive hematopoietic progenitor cells (HPCs) are resistant to HIV-1 infection, once these cells begin to differentiate and become committed HPCs they become increasingly susceptible to HIV-1 infection and permissive to viral gene expression and infectious virus production. Trafficking of BM-derived HIV-1-infected monocytes has been shown to be involved in the dissemination of HIV-1 into the central nervous system (CNS), and it is possible that HIV-1 replication in the BM and infection of BM HPCs may be involved in the early steps leading to the development of HIV-1-associated dementia (HAD) as an end result of this cellular trafficking process. In addition, the growth and development of HPCs in the BM of patients with HIV-1 has also been shown to be impaired due to the presence of HIV-1 proteins and changes in the cytokine milieu, potentially leading to an altered maturation process and to increased cell death within one or more BM cell lineages. Changes in the growth and differentiation process of HPCs may be involved in the generation of monocyte populations that are more susceptible and/or permissive to HIV-1, and have potentially altered trafficking profiles to several organs, including the CNS. A monocyte subpopulation with these features has been shown to expand during the course of HIV-1 disease, particularly in HAD patients, and is characterized by low CD14 expression and the presence of cell surface CD16.

## Introduction

**Figure 1:**
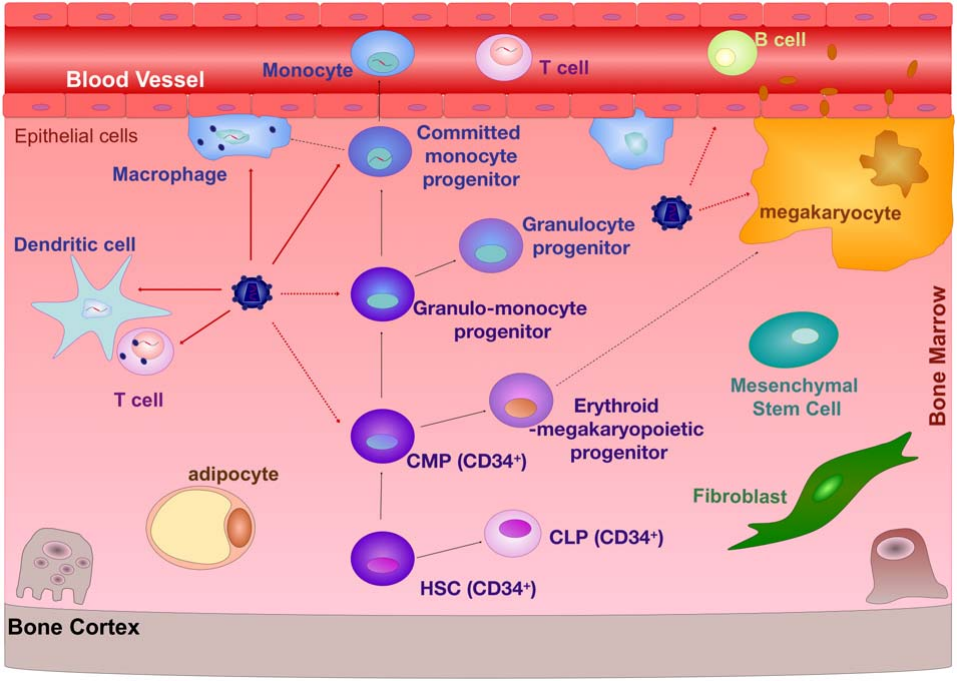
Cells of the BM Susceptible to HIV-1 Infection. The cellular components of the BM include HPCs at all stages of differentiation, megakaryocytes, fibroblasts, endothelial cells, adipocytes, macrophages, osteoblasts, osteoclasts, and MSCs, while DCs, T cells, and B cells may also migrate from the blood into the BM. A black arrow indicates the differentiation process from one cell type to another. A dashed black line indicates that one or more intermediate cell types have been omitted from the respective differentiation process. A red line points to cells that are known to be infected by HIV-1. A dashed red line points to cells that have been shown to be infected by HIV-1, but the extent of their infection and their role in HIV-1 pathogenesis is questionable.

The bone marrow (BM) is the site of hematopoiesis in humans. The cellular components of the BM include fibroblasts, endothelial cells, adipocytes, macrophages, osteoblasts, osteoclasts, reticular cells, and mesenchymal stem cells (MSCs), which are collectively called stromal cells [1], the megakaryocytes, and the hematopoietic progenitor cells (HPCs) (Figure 1). HPCs consist of a heterogeneous population of cells that includes the hematopoietic stem cells (HSCs), which are the most primitive HPCs, capable of producing of blood cell lineages. The loss of one or more developmental potentials of the HSC results in an HPC committed to any number of specific hematopoietic cell lineages. Besides the loss of pluripotency, committed hematopoietic progenitors display a number of characteristics that differ from their parents, including the lack of capacity for self-renewal, a higher fraction of cells traversing the cell cycle, and a change in their surface protein profile [2]. HSCs are able to give rise to common lymphoid progenitors (CLPs) and common myeloid progenitors (CMPs). Part of lymphoid progenitor differentiation, and particularly T cell differentiation, occurs in the thymus. CLPs are capable of differentiating into T cells, B cells, natural killer (NK) cells, and plasmacytoid dendritic cell progenitors [3]. T cells and NK cell progenitors also share a common progenitor often referred to as the T/NK cell progenitor [4]. Myeloid progenitor differentiation occurs exclusively in the BM. The CMPs, which are also referred to as erythromyeloid progenitors, can differentiate into either erythroid/megakaryopoietic progenitors or granulo/monocyte progenitors. Further differentiation leads to commitment to a single lineage such as red blood cell, megakaryocyte, granulocyte, and monocyte-macrophage lineage [5]. Of these cell types, only the megakaryocyte remains in the BM following maturation and generates platelets by fragmentation of the exvaginations of their membrane, while all others emigrate into the blood [2].

Leukopenias and dysplasias that may affect any of the hematopoietic lineages are commonly observed within the population of patients with HIV-1 [6]–[9], even in the absence of opportunistic infections of the BM, neoplasias, or chemotherapeutic treatment, clearly suggesting that HIV-1 infection is associated with dysfunction within the process of hematopoiesis and that these alterations may very well be due to the direct effects of infectious virus and viral proteins in the BM. Numerous studies have reported on a number of defects within the BM progenitor cell populations in HIV/AIDS patients [10]–[18], while primitive [13] and CD34^+^CD4^+^ progenitor cells [11] have been shown to be depleted in individuals with HIV-1 compared to healthy controls. Studies with non-human primates infected with simian immunodeficiency virus (SIV) or simian–human immunodeficiency virus (SHIV) have generated similar results and have emphasized that the suppression of myelopoiesis occurs early in the course of retroviral disease and involves the most primitive CD34^+^ progenitor cells [17],[18].

## Mechanisms of HIV-1-Induced Myelosuppression

**Figure 2:**
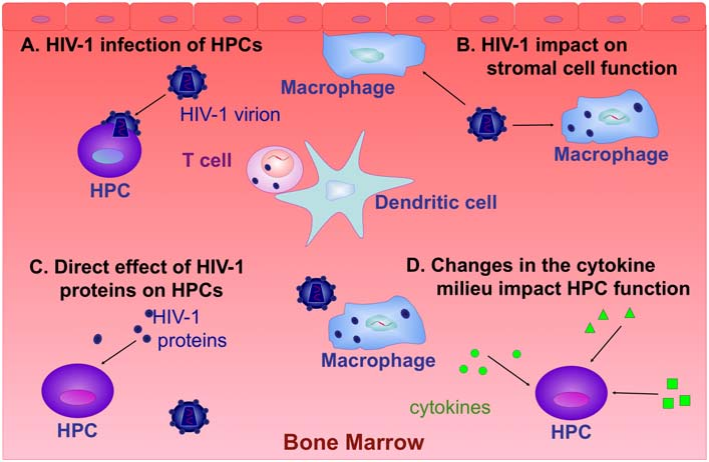
Mechanism of HIV-1-Induced Myelosuppression. Several mechanisms may be involved in HIV-1-induced impairment of hematopoiesis. (A) HPCs may be infected by HIV-1. (B) The interaction of HIV-1 proteins with HPCs may have a direct effect on hematopoiesis. (C) HIV-1 may indirectly affect HPCs by interacting and/or infecting BM stromal cell populations, making them unable to support normal HPC functions. (D) HIV-1 replication in the BM may lead to changes in the cytokine milieu, which may in turn profoundly impact HPC function.

Direct infection of HPCs [19]–[23], inability of stromal cells to carry out their normal functions, toxic effects of HIV-1 proteins, and changes in the cytokine milieu are the most plausible mechanisms for the deleterious consequences of HIV-1 replication in the BM (Figure 2). The BM stroma provides structure, cell-to-cell interactions, and a complex network of inhibitory and stimulatory cytokines that are crucial for the maintenance, differentiation, and proliferation of HPCs. Changes in the BM stromal structure have been observed in patients with HIV-1 and are characterized by a decrease in the fibroblastic population paralleled by an increase of macrophage-like cells [24]. Changes in the clonogenic capacity of BM mesenchymal stem cells, which are responsible for the generation of heterologous stromal lineages including fibroblasts, have been shown [25] and may be responsible for the altered cellular composition of the BM stroma. It has also been suggested that BM microvascular endothelial cells are involved in BM stromal impairment in individuals with HIV-1, exhibiting a decreased capacity to respond to regulatory signals that would normally augment blood cell production [26]. Studies using long-term BM cultures as the in vitro equivalent of the hematopoietic microenvironment have also suggested that BM stroma infected with HIV-1 is unable to support uninfected CD34^+^ progenitor cell expansion and differentiation as compared to uninfected cultures [27]–[29].

A number of reports have indicated that heat-inactivated HIV-1 can induce effects on progenitor cells similar to those induced by fully infectious virus [30],[31]. This observation has prompted investigators to look further into the possible effects of individual HIV-1 proteins on hematopoiesis. Heat-inactivated HIV-1 and cross-linked envelope glycoprotein gp120 were shown to induce a decrease in clonogenic capacity and induce apoptosis through a Fas-dependent mechanism [32],[33]. In contrast, an earlier study had suggested that gp160 may induce a T cell–mediated stimulation of HPCs that could lead to hypercellularity in the BM [34]. Moreover, the HIV-1 Gag protein has been shown to suppress colony formation from HPCs [35], while Vpr was shown to induce phagocytosis of cells in the BM by mononuclear phagocytes, possibly contributing to the failure of HPC colonies to expand [36].

Additionally, Tat has been shown to have a role in myelosuppression through induction of the growth inhibitory cytokine TGF-β [37]. Blocking TGF-β in purified CD34^+^ peripheral blood cells that were exposed to HIV-1 either in vivo or in vitro resulted in a significant improvement in their growth and survival, suggesting a critical role for this cytokine in this important biological process [32]. In agreement with these results, a more recent study reported that gp120 can lead to TGF-β1 upregulation in CD34^+^ HPCs, and at the same time downregulation of APRIL, a cytokine known to induce proliferation [31]. On the contrary, studies investigating TGF-β production within the BM stroma failed to identify any differences in the absence or presence of HIV-1 [26],[30],[38]. This observation points to the inherent difficulty in examining the effect of individual proteins on HPCs—that is, whether the extracellular concentration of HIV-1 proteins in the BM is enough to promote the same effects as observed in vitro.

Several hematopoietic growth factors, as well as cytokines associated with inflammation and immune activation, have been investigated with respect to interference in HPC expansion. A role for stem cell factor (SCF), granulocyte-macrophage-colony stimulating factor (GM-CSF), and IL-3 has not been observed [26],[38], while an impairment in granulocyte–colony stimulating factor (G-CSF) and IL-6 production from stromal cells has only been shown following IL-1α stimulation [26]. IFN-α and IL-4 have been reported to be responsible for part of the HIV-1-induced myelosuppression observed in long-term BM cultures [29]; however, results from other studies are in disagreement with this result [26],[30]. Previous studies have reported a decrease in IL-2 production from BM stromal cells derived from patients with HIV-1, but a direct correlation with HPC impairment has not been demonstrated [24],[39]. The proinflammatory cytokines TNF-α, IL-1α, and IL-6, as well as the β-chemokines MIP-1α, MIP-1β, and RANTES, have been reported to be upregulated in the BM of patients with HIV-1 [24],[25],[39], while TNF-α involvement in HIV-1-induced suppression of hematopoiesis has been shown in neutralization studies [30],[40]. SDF-1, the natural ligand for CXCR4, is constitutively present in the BM [41], but there are no reports examining changes in SDF-1 expression in the BM of patients with HIV-1. Nevertheless, SDF-1 has been shown to regulate adhesion survival and proliferation of HPCs [42], and its role in the HIV-1-infected BM warrants investigation, as it may be involved in limiting infection by CXCR4-utilizing strains of HIV-1.

## HIV-1 Infection of Accessory Cells of the BM

Among the stromal cells, macrophages are the only, or the most prominent, cell type that are productively infected with HIV-1 and express viral antigens both in vivo [24] and in primary BM cultures [43],[44]. Importantly, macrophages in the BM were shown to be susceptible to both macrophage tropic and T cell–tropic strains of HIV-1, and to have a broader susceptibility to HIV-1 strains than blood-derived macrophages [44]. Consistent with the human studies, results from SIV-infected macaques suggest that macrophages are the primary target of SIV within the BM compartment [45]. Eosinophils differentiated in vivo from BM stromal cells, and BM fibroblasts, have been reported to be permissive for HIV-1 [46],[47]. However, these results have not been confirmed by more recent reports, and contamination of the eosinophils and fibroblast cultures with macrophages may have contributed to the results observed in these studies. Limited susceptibility of endothelial cells to HIV-1 has been demonstrated in various systems, and BM microvascular endothelial cells devoid of macrophages have been shown to be the predominant cells infected in BM stromal cultures [26].

Megakaryocytes, as well as their precursors, express CD4 [6],[48] and CXCR4 [49],[50], and several studies have shown that they are permissive for HIV-1 infection [51]–[53]. Notably, in a more recent study, in addition to CD4 and CXCR4, CCR5 was detected on the surface of megakaryocytes, and infection of these cells with both CCR5-tropic and CXCR4-tropic HIV-1 has been reported [53]. It has been postulated that thrombocytopenia, which is a very common finding in patients suffering from HIV disease, may be due to infection of megakaryocytes; however, in the absence of productive infection, HIV-1 has been shown to negatively affect the survival and maturation of HPCs towards megakaryocytes, and it has been suggested that gp120–CD4 interaction may be mediating the observed effect [31].

## HIV-1 Infection of HPCs

The literature regarding susceptibility of BM stem cells and HPCs to HIV-1 infection is extensive, and although it has been almost 20 years since the first report of HPC infection [54], there is still considerable controversy in this area of investigation. Early studies have shown that CD4 is expressed in 25%–65% of CD34^+^ BM cells, and these studies have estimated that the level of expression is half of what has been observed in monocytes and only 5% of the level reported on CD4^+^ T cells [55],[56]. Several studies have confirmed these results and have also demonstrated the presence of CXCR4 and CCR5 on these cells. The results have been rather consistent regardless of the source of CD34^+^ cells or the state of differentiation, and whether or not mRNA or protein levels were assessed [11], [23], [57]–[60]. The main difference among these studies has concerned the level of CCR5 expression, which appears to vary depending on the state of differentiation of the progenitor cell population [23].

Despite the detection of the HIV-1 receptor and coreceptors on HPCs, in vitro studies designed to infect these cells with HIV-1 have generated conflicting observations. The first report to describe HIV-1 infection of HPCs was able to detect infected cells 1 to 2 months after virus exposure, when the cells had stopped expressing HPC markers and had become lineage-specific cells [54]. This study, although a landmark investigation, has been challenged with respect to the purity of the initial CD34^+^ BM culture used to establish the infected cell population [6],[61]. Later studies that demonstrated HIV-1 infection of HPCs were also performed with mixed CD34^+^ populations prepared from the BM, which included early progenitors along with some more differentiated and lineage-committed CD34^+^ cells [19],[20]. HIV-1 infection studies performed on peripheral blood–derived CD34^+^ cells generated similar results but also failed to address the purity and differentiation state of the initial CD34^+^ target cell population [21]. To determine the major cell type in the mixed CD34^+^ BM population susceptible to HIV-1, cells were cloned in solid cultures [22] and the presence of virus in colony-forming units (CFU) was determined. Granulocyte-macrophage-CFU and to a lesser extent erythroid-burst forming units were infected; however, granulcyte-erythroid-macrophage-megacaryocyte-CFU were not, indicating that some primitive HPCs, but not the multipotent HPCs, may be susceptible to in vitro HIV-1 infection. When the AC133 marker was used to purify the most primitive CD34^+^ cells from cord blood, infection with HIV-1 was not possible [62]. Similarly, the CD38 marker has been used in some studies to define the differentiation state of CD34^+^ HPCs. CD38 is absent from the early HPCs and appears as they differentiate, but before they start expressing lineage-specific markers and downregulating CD34 expression [63]. Weichold et al. showed that CD34^+^/CD38^+^ BM cells were susceptible to both M-tropic HIV-1 and HIV-2, but they were not able to conclusively demonstrate infection of the more primitive CD34^+^/CD38^−^ cells with either type of virus [64]. However, an additional study has reported that both CD34^+^/CD38^+^ and CD34^+^/CD38^−^ cells were susceptible to HIV-1, and that only the very early stem cells, which are metabolically inactive and arrested at the G_0_ state, were resistant to HIV-1 [23].

Although in vitro HIV-1 infection of HPCs has been shown in a number of studies, detection of HIV-1-infected HPCs in vivo has only been demonstrated within a subpopulation of patients. In 1990, von Laer et al. reported that only one in 14 patient samples of BM-derived CD34^+^ cells harbored proviral DNA [65]. However, the sensitivity of the PCR assay used in this study may be questionable since in the same study HIV-1-specific sequences were detected within the monocytes of two of the 14 patients. However, consistent with these observations, a highly sensitive PCR assay that was shown to detect one copy of HIV-1 in 10^3^ cells was used by Davis et al. to demonstrate the presence of proviral DNA in BM-derived CD34^+^ cells in only one out of 11 patients with HIV-1 [66]. Much higher frequencies of HIV-1 infection of CD34^+^ cells were reported within Zairian patients with HIV disease (36.5%). In the same study, 14% of patients infected with HIV-1 from the United States appeared to harbor viral sequences in their CD34^+^ cell population [67], a frequency marginally higher than that described in earlier reports. Higher frequencies of viral infection within the BM CD34^+^ cell compartment have been reported in more recent studies, ranging from 20% to 28% [14],[61],[68], possibly reflecting an improvement in the sensitivity of the PCR assays used. Nevertheless, when interpreting these studies, the purity of the CD34^+^ population should be considered, given that the BM is highly vascular and it is impossible to distinguish whether the virus is associated with resident cells of the BM or with cells present in the vasculature.

Overall, the spectrum of in vitro and in vivo studies points to the fact that HIV-1 infection of the HPC compartment is limited in extent. The refractile nature of this developing immune cell precursor population, despite the expression of CD4, CCR5, and CXCR4, may be explained by a potential masking of the gp120 binding site. In fact, CD34^+^ HPCs have been shown to secrete the CCR5 ligands MIP-1α, MIP-1β, and RANTES [69],[70], and the CXCR4 ligand SDF-1 [60], which may compete with infectious HIV-1, either CCR5- or CXCR4-utilizing virus, and consequently block infection.

The difficulty in detecting HIV-1-infected HPCs in vivo may also be due to the limited time frame during which these cells express progenitor cell markers following their infection with HIV-1. A number of studies suggest that the HPCs that are susceptible to HIV-1 are not the most primitive ones and they may already be committed to a cell lineage. Infection of megakaryocyte progenitors [71] and mast cell progenitors [72],[73] by HIV-1 has been demonstrated, while conditions that induce differentiation towards dendritic cells (DCs) [74] and monocytes have been shown to promote infection of HPCs [20],[57]. Therefore, it is likely that the HPC that gets infected with HIV-1 can only undergo a limited number of cell divisions and is destined to promptly lose its progenitor surface markers. Moreover, HIV-1 infection of HPCs may promote their differentiation and decrease the time frame in which these cells may be identified as HPCs.

Indirect evidence that HPCs infected with HIV-1 can survive and proliferate was derived from a study where a defective HIV-1 sequence had been integrated in the genome of both CD8^+^ T cells and granulocytes. Since the provirus was unable to guide multiple rounds of infection, it was assumed that HIV-1 infection must have occurred in a common progenitor of CD8^+^ T cells and granulocytes [75]. However, given the very small pool of HPCs that may be infected with HIV-1, and possibly in only a limited number of patients, it is often implied that HIV-1 infection of HPCs is physiologically unimportant [61],[65],[66]. Radically contradicting this line of thinking, a recent study argues that even one or two infection events of a key cell population with proliferative capacity and long-term persistence may be a crucial obstacle for eradication. Bailey et al. demonstrated that in some patients with HIV-1 undergoing successful highly active antiretroviral therapy (HAART) there are one or two predominant viral sequences in their plasma over prolonged periods of time. These sequences differ from those commonly found in CD4^+^ T cells, and do not carry any known resistance mutations, which suggests that the rare infection of a small number of progenitor cells is the most likely explanation for the observed residual viremia [76].

## Trafficking of HIV-1-Infected Cells to and from the BM

The BM, in addition to its role in the generation of all hematopoietic cell lineages, is also a primary and a secondary lymphoid organ, and therefore is a site for the continuous interaction of immune cells, which provides an expanded opportunity for the dissemination of HIV-1. The role of the BM as a secondary lymphoid organ has been emphasized by recent evidence indicating that the BM serves as a major reservoir of central memory T cells [77] while it also recruits naïve T cells [78] and promotes antigen-presenting cell and T cell interaction [79],[80]. Importantly, it has been shown that CD4^+^ T cells in the blood of patients with HIV migrate to the BM at rates higher than those in uninfected individuals, and that the enhanced homing of these cells correlates with active HIV-1 replication [81]. Studies in the SIV-macaque model of HIV-1 infection have shown dissemination of the virus into the BM within the first 2 weeks following inoculation [82]. As CD4^+^ T cells migrate into the BM, they may provide a source of replicating virus and be responsible for the initial dissemination of the virus into this anatomical compartment. However, CD4^+^ T cells are not the only possible candidates for introduction of HIV-1 into the BM; DCs emigrating from tissues into the peripheral circulation have been shown to traffic to the BM, where they were retained and able to interact with central memory T cells and trigger immune responses [79]. Although possible, this route of DC trafficking has not yet been shown in HIV-1 or SIV models; instead, DCs have been shown to be infected with SIV in mucosal tissues and to disseminate through the lymphatic circulation in the draining lymph nodes [83],[84]. In addition to cell-associated virus, cell-free virus may also be involved in the dissemination of HIV-1 within the BM compartment.

**Figure 3:**
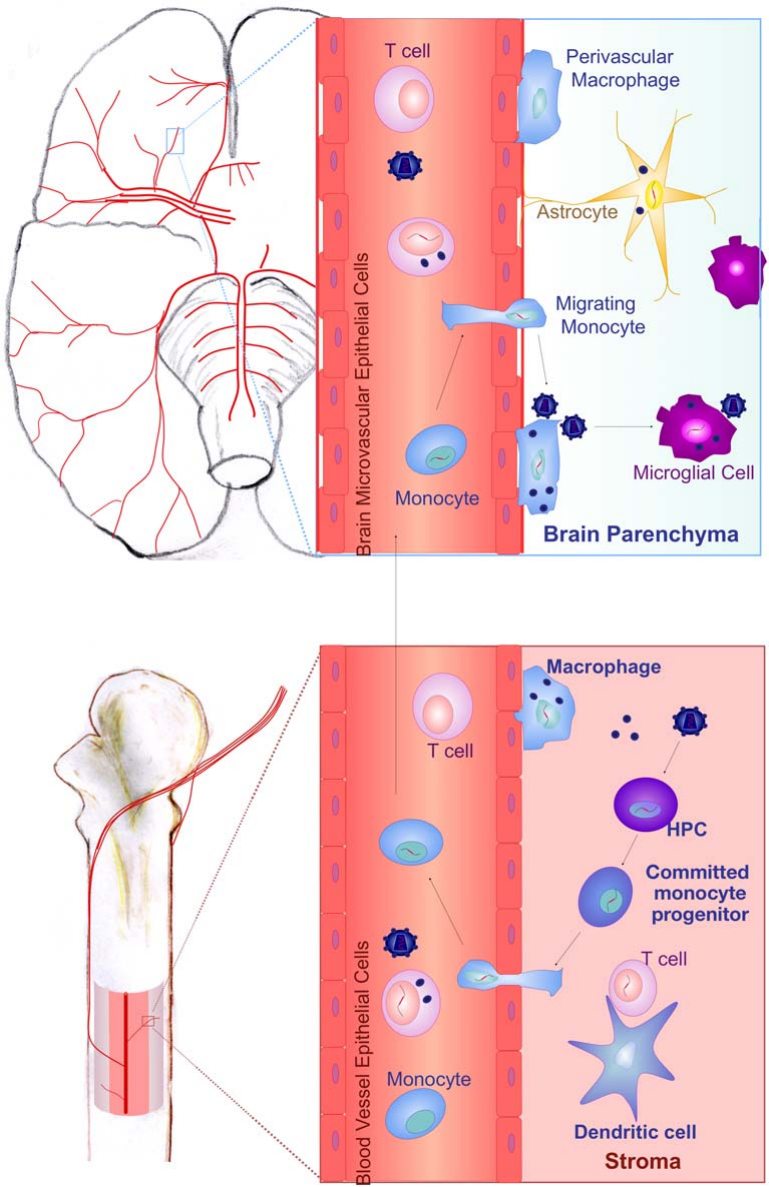
Trafficking of HIV-1-Infected Cells from the BM to the CNS. Monocytes infected in the BM with HIV-1 either as HPCs or as commited monocyte progenitors emigrate from the BM into the blood, and through the blood circulation reach the capillary vessels of the brain, where they may cross the blood–brain barrier and migrate into the CNS. Following entry into the CNS, monocytes differentiate into perivascular macrophages, which has been shown to typically lead to upregulation of viral protein production, release of virions, and infection of resident cells of the CNS.

Once HIV-1 has established a productive infection in the BM, opportunity for dissemination considerably increases since cells originating from the BM, primarily monocytes, are able to travel to all tissues. Monocytes may also emigrate from the BM uninfected and become infected by HIV-1 during their circulation in the blood. Considering the relative resistance of monocytes to HIV-1, due to the expression of APOBEC3G [85], it is currently unknown what is the relative contribution of monocyte and monocyte progenitor infection to the pool of infected circulating monocytes in patients with HIV-1. Lungs, gastrointestinal tract, kidney, urogenital tract, primary and secondary lymphoid organs, and the central nervous system (CNS) are some of the organs that BM-derived monocytes will migrate into [86], and these monocytes will differentiate toward tissue-specific macrophages and DCs. Migration of HIV-1-infected monocytes into the lungs may play an important role in the pathophysiology of pulmonary disease [87]. Migration of infected cells of the monocyte-macrophage lineage into the mucosal tissues of the gastrointestinal tract or the urogenital tract is thought to play a critical role in viral transmission [88]. However, the most profound impact of HIV-1-infected monocyte trafficking is caused by their ability to cross the blood–brain barrier and deliver HIV-1 into the CNS (Figure 3), contributing not only to HIV-1-associated neurologic dysfunction but also to the life-long persistence of HIV-1. A continuous repopulation of macrophages, and to a lesser extent microglia, occurs in the uninfected CNS, and has been shown to be considerably accelerated in cases of inflammation [89]. Studies in rodents have shown that approximately 60%, 30%, and 1% of meningeal macrophages, perivascular macrophages, and microglia, respectively, are replaced by cells originating from the BM within a 1-month period [90]. During the repopulation of macrophages and microglia from BM-derived monocytes, some of which may be infected with HIV-1, the virus gains access to the CNS. In support of this model, an elegant study has recently reported monocyte migration into the CNS in acute SIV infection coupled with concurrent dissemination of the virus to this compartment [91]. Although CD4^+^ T cell trafficking in the CNS also occurs, its contribution to the dissemination of HIV-1 in the CNS has not been characterized. Interestingly, it has been shown that the majority of viral sequences in the cerebrospinal fluid are generated by short-lived cells, possibly CD4^+^ T cells, that have been infected inside the CNS [92], emphasizing that CD4^+^ T cells may have a role in viral dissemination inward but also outward of the CNS. The possibility of cell-free virus or trafficking into the CNS has not been excluded, but is considered of lesser importance in the pathogenesis of HIV-1-associated CNS disease [93]–[96].

Two main monocyte subsets have been described, the CD14^hi^/CD16^−^ monocytes, which are the most prevalent, and the CD14^low^/CD16^+^ monocytes, which consist of approximately 5%–15% of circulating monocytes in healthy individuals. In a number of pathologic conditions this monocyte population has been shown to expand [97],[98], and in patients with HIV-1, the CD14^low^/CD16^+^ monocyte population may increase to as high as 40% of the total circulating monocyte population [99]. It is unclear whether this expansion is due to the increased production of CD14^low^/CD16^+^ monocytes in the BM or whether it occurs after differentiation of the CD14^hi^/CD16^−^ to CD14^low^/CD16^+^ monocytes and represents a different state of maturation [100],[101]; in either case, this monocytic subset has been shown to exhibit features that link it to the development of HIV-1-associated CNS disease. Studies have shown that the CD14^low^/CD16^+^ monocytes have a more activated, proinflammatory phenotype [102], and that they are increased in patients with HIV-1-associated dementia (HAD) more than they are in non-demented patients with HIV-1 [103]. Recently, the CD14^low^/CD16^+^ monocyte was shown to be more susceptible to HIV-1 entry and permissive to productive infection [104]. These cells are thought to migrate more effectively to the CNS than the classical CD14^hi^/CD16^−^ monocytes and have been shown to accumulate in perivascular regions of the brain, where they have also been shown to colocalize with HIV-1-specific proteins [105],[106].

Currently, there are no definitive direct observations demonstrating that the monocytes migrating to the brain have been infected with HIV-1 while they were in the BM, potentially as monocyte progenitors, or whether infection occurred while in the blood. However, HIV-1-specific sequences from the BM resemble sequences from the deep white matter of the brain more than any other tissue, suggesting direct HIV-1 trafficking from the BM into the CNS [107], at least during the later stages of HIV-1 disease. However, it is possible that HIV-1 infection in the BM, which causes changes in the cytokine milieu as previously described, impacts the maturation of HPCs towards the monocytic lineage, resulting in the generation of a monocyte subset that is more prone to migration into tissues and specifically into the CNS. This monocyte subset, possibly represented by the CD14^low^/CD16^+^ phenotype, is more susceptible to infection and becomes infected by HIV-1 before it leaves the BM or shortly thereafter, thus contributing to viral dissemination. Importantly, anemia [108], as well as thrombocytopenia [109], has been shown to correlate tightly with the development of HAD, suggesting that HIV-1-induced pathogenesis of the BM may impact HAD progression. Similarly, a recent report showing that BM diffusion rates correlate with dementia severity [110] provides additional support for the hypothesis that events in the BM may be associated with the development of HAD [95].

## Conclusions

The central role of the BM in the pathogenesis of HIV-1 is highlighted by the wide spectrum of hematopoietic abnormalities observed in patients with HIV-1. It is has been questioned whether any of these abnormalities are due to direct HIV-1 infection of HPCs; however, collectively the data suggest that the presence and replication of HIV-1 in the BM impact either directly or indirectly the normal proliferation and differentiation of HPCs, resulting in changes in the cell populations of the blood. The effect on the generated monocytes may not be solely in numbers but also in their migratory properties and in their susceptibility to HIV-1, ultimately impacting the pathogenesis of AIDS and HAD.
